# Comparative Metabolomics and Molecular Phylogenetics of Melon (*Cucumis melo*, Cucurbitaceae) Biodiversity

**DOI:** 10.3390/metabo10030121

**Published:** 2020-03-24

**Authors:** Annick Moing, J. William Allwood, Asaph Aharoni, John Baker, Michael H. Beale, Shifra Ben-Dor, Benoît Biais, Federico Brigante, Yosef Burger, Catherine Deborde, Alexander Erban, Adi Faigenboim, Amit Gur, Royston Goodacre, Thomas H. Hansen, Daniel Jacob, Nurit Katzir, Joachim Kopka, Efraim Lewinsohn, Mickael Maucourt, Sagit Meir, Sonia Miller, Roland Mumm, Elad Oren, Harry S. Paris, Ilana Rogachev, Dominique Rolin, Uzi Saar, Jan K. Schjoerring, Yaakov Tadmor, Galil Tzuri, Ric C.H. de Vos, Jane L. Ward, Elena Yeselson, Robert D. Hall, Arthur A. Schaffer

**Affiliations:** 1 INRAE, Univ. Bordeaux, UMR1332 Fruit Biology and Pathology, Bordeaux Metabolome Facility MetaboHUB, Centre INRAE de Nouvelle Aquitaine - Bordeaux, 33140 Villenave d’Ornon, France; annick.moing@inrae.fr (A.M.); bbiais@free.fr (B.B.); catherine.deborde@inrae.fr (C.D.); daniel.jacob@inrae.fr (D.J.); mickael.maucourt@inrae.fr (M.M.); dominique.rolin@inrae.fr (D.R.); 2 The James Hutton Institute, Environmental & Biochemical Sciences, Invergowrie, Dundee, DD2 5DA Scotland, UK; william.allwood@hutton.ac.uk; 3 Department of Plant and Environmental Sciences, Weizmann Institute of Science, Rehovot 7610001, Israel; asaph.aharoni@weizmann.ac.il (A.A.); sagit.meir@weizmann.ac.il (S.M.); Shifra.ben-dor@weizmann.ac.il (S.B.-D.); 4 Rothamsted Research, Harpenden, Herts AL5 2JQ, UK; john.baker@pfizer.com (J.B.); mike.beale@rothamsted.ac.uk (M.H.B.); sonia.miller83@yahoo.co.uk (S.M.); jane.ward@rothamsted.ac.uk (J.L.W.); 5 Max Planck Institute of Molecular Plant Physiology, Potsdam-Golm 14476, Germany; federicobrigante@outlook.com (F.B.); Erban@mpimp-golm.mpg.de (A.E.); Kopka@mpimp-golm.mpg.de (J.K.); 6 Universidad Nacional de Córdoba, Facultad de Ciencias Químicas, Dto. Química Orgánica, Córdoba 5000, Argentina; 7 CONICET, ICYTAC (Instituto de Ciencia y Tecnologia de Alimentos Córdoba), Córdoba 5000, Argentina; 8 Institute of Plant Science, Agricultural Research Organization—Volcani Center, Rishon LeZiyyon 7515101, Israel; burgery@agri.gov.il (Y.B.); adif@agri.gov.il (A.F.); elenae@agri.gov.il (E.Y.); 9 Newe Ya‘ar Research Center, Agricultural Research Organization, P. O. Box 1021, Ramat Yishay 3009500, Israel; amitgur@volcani.agri.gov.il (A.G.); katzirn@agri.gov.il (N.K.); twefraim@agri.gov.il (E.L.); elad.oren@mail.huji.ac.il (E.O.); hsparis@agri.gov.il (H.S.P.); yarden@agri.gov.il (U.S.); tadmory@agri.gov.il (Y.T.); galilt@agri.gov.il (G.T.); 10 Department of Biochemistry, Institute of Integrative Biology, University of Liverpool, Liverpool L69 7ZB, UK; roy.goodacre@liverpool.ac.uk; 11 Department of Plant and Environmental Sciences & Copenhagen Plant Science Center, Faculty of Science, University of Copenhagen, DK-1871 Frederiksberg C, Denmark; thh@plen.ku.dk (T.H.H.); jks@plen.ku.dk (J.K.S.); 12 Business Unit Bioscience, Wageningen University & Research, Post Box 16, 6700AA, Wageningen, Netherlands; roland.mumm@wur.nl (R.M.); robert.hall@wur.nl (R.D.H.); 13 Department of Plant Physiology, Wageningen University & Research, Laboratory of Plant Physiology, Post Box 16, 6700AA, Wageningen, Netherlands

**Keywords:** genetic resources, melon, genotype by sequencing, elemental analysis, metabolome, *Cucumis melo*

## Abstract

The broad variability of *Cucumis melo* (melon, Cucurbitaceae) presents a challenge to conventional classification and organization within the species. To shed further light on the infraspecific relationships within *C. melo*, we compared genotypic and metabolomic similarities among 44 accessions representative of most of the cultivar-groups. Genotyping-by-sequencing (GBS) provided over 20,000 single-nucleotide polymorphisms (SNPs). Metabolomics data of the mature fruit flesh and rind provided over 80,000 metabolomic and elemental features via an orchestra of six complementary metabolomic platforms. These technologies probed polar, semi-polar, and non-polar metabolite fractions as well as a set of mineral elements and included both flavor- and taste-relevant volatile and non-volatile metabolites. Together these results enabled an estimate of “metabolomic/elemental distance” and its correlation with the genetic GBS distance of melon accessions. This study indicates that extensive and non-targeted metabolomics/elemental characterization produced classifications that strongly, but not completely, reflect the current and extensive genetic classification. Certain melon Groups, such as Inodorous, clustered in parallel with the genetic classifications while other genome to metabolome/element associations proved less clear. We suggest that the combined genomic, metabolic, and element data reflect the extensive sexual compatibility among melon accessions and the breeding history that has, for example, targeted metabolic quality traits, such as taste and flavor.

## 1. Introduction

*Cucumis melo* L., melon, is a phenotypically highly variable species with respect to fruit characteristics [1]. Melon fruits vary not only in size and shape but also in the accumulation of various metabolites, the most obvious of which include the horticulturally important metabolites of external and internal pigmentation, volatiles responsible for fruit aroma, and carbohydrates and organic acids accounting for sweetness and acidity [2]. Melon fruit range in sizes up to 20 kg; in shape from spherical to very long; in taste from insipid to sweet, acidic, or bitter; have external colors of green, yellow, orange, and red, with internal flesh colors of white, green, orange, or cream; and encompass a broad range, from highly aromatic to almost non-aromatic types. The species is unique in that it has representatives of both climacteric and non-climacteric ripening physiology [3], which further impacts on the metabolite components of the ripe fruit.

The extreme fruit variation within *Cucumis melo* does not easily lend itself to conventional infraspecific classification. Kirkbride [4] suggested that the species should be considered to consist of two subspecies, based on the pubescence of ovaries and young fruits. Accordingly, ovaries and young fruits having appressed short hairs were assigned to *C. melo* subspecies *agrestis* and those having pilose or lanate, spreading, long hairs were assigned to *C. melo* subsp. *melo*. However, it is often not easy to reconcile young fruit pubescence with the melon fruits seen in marketplaces in various parts of the world. Although markets in some regions feature young cucumber-like melons, most markets feature mature ripe sweet dessert melons, others ripe and highly aromatic but insipid “duda’im” melons, and yet others fully grown but unripe “snap” melons [1,5].

Attempts to classify melons according to fruit characteristics date to the first half of the 19th century, and over the past two centuries, a great number of proposed infraspecific classifications for melons have accumulated (reviewed in [6]). Among these proposed classifications are “lumpers”, which have not indicated enough divisions within the species to adequately reflect its wide variation, and “splitters”, which have fragmented the species into an unwieldy number of units. To be feasible, any useful infraspecific classification must at once be reflective of genetic relationships and also be easily observed by those for whom the classification is intended, which should be a wide international audience that includes scientists and non-scientists alike. For melons, this means that the characteristics of fruits should serve as the basis for any such classification. Given all of the above considerations, our view is that the infraspecific breakdown to 16 varietas first proposed by Pitrat et al. [6] or cultivar-groups (“Groups”) (adopted by Burger et al., [2]) is currently the most useful. Groups are lower ranking than the subspecies, but each Group can consist of more than one market type.

The economically most important Groups are the Reticulatus (climacteric, netted), Cantalupensis (climacteric, non-netted), Inodorus (non-climacteric), and Ameri (climacteric, dryland cultivation, Asian), all of which have sweet flesh when ripe and belong to subsp. *melo*. Other Groups include the Flexuosus (snake melon), Duda’im (pocket melon), Momordica (snap melon), and Khandalak, and the East Asian Conomon (Oriental pickling), Makuwa, and Chinensis Groups. However, even the placement of the various Groups into the two subspecies is not fully agreed upon, as may be expected from a somewhat subjective classification based on the single trait of ovary pubescence. The recent attempts at making order within the melon species, at the subspecies level, literally focused on hair-splitting. For example, while the *agrestis* subspecies proposed by Pitrat et al. [6] encompasses five Groups (Conomon, Makuwa, Chinensis, Momordica, and Acidulus), the *agrestis* as described by Decker-Walters et al. [7] combines Momordica, Conomon, Duda’im, and Chito. The latter two Groups, Duda’im and Chito, were placed in subsp. *melo* by Pitrat et al. [6], and Zhao et al. [8] recently traced the Momordica Group to subsp. *melo*. Furthermore, the distinction between Reticulatus (climacteric, netted) and Cantalupensis (climacteric, non-netted) based on netting is particularly unclear since the netting phenotype is expressed as a continuum of what is basically a quantitative trait. Thus, semi-netted Charentais-type melons have vacillated between the two infraspecific Groups [6,9,10].

The difficulty of clear infraspecific classification of the species is further confounded by the ease of crossbreeding within the species, as well as the undocumented history, both early and more recent, of most accessions, and the widespread occurrence of feral melons. Even the natural range of melon species has been the subject of ongoing studies. Traditionally, melon has been grouped with other *Cucumis* spp. associated with African origin [4]. However, recent studies indicate a closer relationship to an Australian/Asian clade, suggesting that *C. melo* has origins in Asia [11,12]. Inodorous Group melons have a Central Asian origin, traceable to the mid-9th century and had arrived in Spain by the 11th century [13]; Duda’im Group melons have been traced to mid-9th century Persia [14]; and Flexuosus Group melons have been traced back 4000 years to ancient Egypt [15,16].

Molecular-based phylogenetics contributed to the infraspecific classification of *C. melo*, consistently supporting a subspecies separation, with small differences in the placement of some of the Groups [17,18]. Recently, Endl et al. [19] pointed to the complexity within the *agrestis* subspecies and showed that there is likely a complex African/Asian/Australian origin of the *agrestis* types of *C. melo*, based on polymorphisms in seven DNA regions. The most recent and encompassing phylogenetic classification of the species was reported by Zhao et al. [8], which corroborated the subspecies classification but placed the Indian Momordica Group within the *melo* subspecies. Interestingly, Leida et al. [20], based on ca. 200 single nucleotide polymorphisms (SNPs), identified what they refer to as Spanish and European Inodorus Group landraces within the *agrestis* subspecies, distinct from the well-known cultivated Inodorus Group, suggesting that the non-climacteric genetic trait may have evolved independently multiple times.

Metabolomic-based phylogeny, or chemosystematics, has regularly been attempted from the early part of the last century [21], and was especially popular in the 1970s. However, these studies focused on intra-family classification at the species level and were generally based on the measurement of individual components of single biochemical families, frequently alkaloids, as allowed by the technologies of the period. For example, the chemical systematics of the family Rutaceae and the order Rutales attracted much research attention. This chemosystematic classification has been compared to the phylogeny determined by molecular polymorphism strategies and found to be generally confirmatory [22]. However, chemical systematics has not been attempted at the infraspecific level for the obvious reason that a limited number of chemicals are unlikely to be adequate for describing the extent of varietal differences. Furthermore, relatively minor differences due to few, or even single, genes may still be causal of large differences in metabolic components. For example, the genetic polymorphism controlling ripening physiology in melons, whether climacteric or non-climacteric, although essentially a simply inherited trait [3], will likely be accompanied by major changes in the primary and specialized metabolomes.

While small subsets of metabolites may not be useful for infraspecific systematics and phylogenetics, the number of metabolites available that can be analyzed by large-scale metabolomic strategies has potential discriminatory power. The advances in metabolomics technologies now allow for these techniques to be used and to attempt to analyze infraspecific relationships [23]. Recently, we described a metabolomic comparison of two melon cultivars [24], and the results were encouraging regarding the relevance of such analyses to a larger infraspecific classification. Furthermore, we performed a targeted metabolomic characterization of 77 metabolites, primarily of quality-determining volatiles, sugars, and carotenoids, of a novel segregating genetic population derived from random introgressions between two distantly related melon accessions [25]. The results indicated the potential of metabolomic analyses of genetic variability for the discovery of associations between metabolites and metabolic pathways. Esteras et al. [26] also recently surveyed the volatile and carotenoid profiles of broad genetic variability of melon and emphasized the value of unidentified and untapped diversity for melon fruit quality improvement.

Considering the presence of hundreds of thousands of primary and specialized metabolites in the plant kingdom, no single metabolomics platform is capable of describing the plant metabolome; however, the combination of multiple platforms can approach that goal. In this study, the metabolite profile of 51 melon accessions, representing the broad genetic variability in the species, was characterized in depth using an orchestra of metabolomic strategies. The metabolomic technologies used comprised flow injection electrospray mass spectrometry (FIE-MS) fingerprinting of semi-polar extracts, untargeted proton nuclear magnetic resonance (^1^H-NMR) profiling of polar or semi-polar extracts, liquid chromatography coupled to QTOF (quadrupole time-of-flight) mass spectrometry (LC-QTOF-MS) of semi-polar compounds, gas chromatography coupled to mass spectrometry (GC-MS) of polar extracts, solid-phase microextraction (SPME) GC-MS of volatiles, and inductively coupled plasma mass spectrometry (ICP-MS) of mineral elements [27]. This broad array of technologies collected over 80,000 metabolite signatures, i.e., molecular features.

The analyses detailed in this report are based on the comparison of the infraspecific classification as determined by metabolomic profiling of an extensive collection of accessions representing most of the cultivar-groups of *Cucumis melo*, with the classification derived from extensive comparative genotyping. The unique combination of >80,000 specific molecular features and >20,000 genetic polymorphisms obtained in this study allowed us to compare the inferred infraspecific phylogenies derived from each of the strategies and to test the relationships between metabolomic and genetic distance in this complex widely cultivated species.

## 2. Results

### 2.1. Infraspecific Structure of Cucumis melo

**Figure 1:**
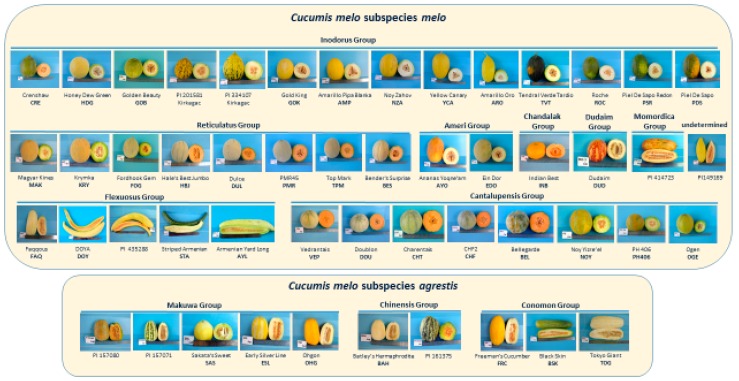
Fruits of the accessions used for this study, representing most of the cultivar-groups of *Cucumis melo* (listed in Table 1 and Table S1): Cantalupensis, Reticulatus, Inodorus, Ameri, Flexuosus, Dudaim, Momordica, Khandalak, Conomon, Chinensis, and Makuwa.

Fifty-one accessions of *Cucumis melo*, representing the cultivar-groups Reticulatus, Cantalupensis, Inodorus, Ameri, Flexuosus, Duda’im, Momordica, Khandalak, Conomon, Makuwa, and Chinensis, were grown in Israel during a spring-summer season and flesh and rind samples of mature fruit were harvested and distributed to the collaborating laboratories for the respective metabolomics analyses (Figure 1, Table 1; Table S1). In our classification into subspecies and cultivar-groups, we followed the proposed classifications of Pitrat et al. [6] and Burger et al. [2], with the exception of the placement of the single Momordica accession PI414723, which we included among the subspecies *melo*. 

In parallel, 44 accessions could be genotyped by sequencing (GBS), which, in brief, is based on high-throughput sequencing of restriction enzyme fragments of sample DNA. For the present study, GBS provided 23,931 informative SNPs, which were selected for genome-wide analyses and phylogenetic classification. The accessions not included in the GBS analysis were provisionally genotyped by direct sequencing of over 11,000 bps, derived from 20 PCR reactions, yielding 116 informative SNPs (Figure S1).

**Figure 2:**
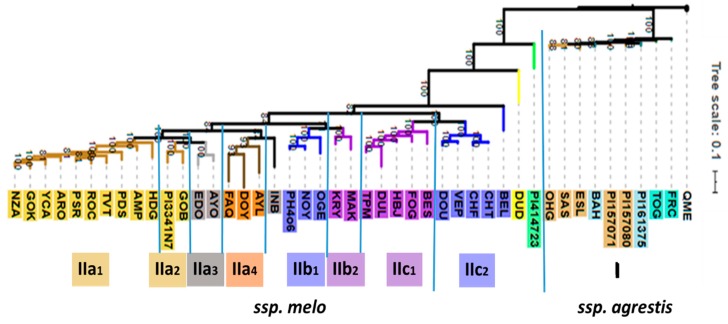
Phylogram representing the phylogenetic relationships of the 44 accessions of *C. melo* used for GBS analysis in this study. The feral QME serves as outlier. Bootstrap values are based on 100 iterations.

Based on 23,931 genetic polymorphisms, the 44 accessions could be classified into two well-defined clusters (referred to as I and II) clearly distinguishing between the subspecies *agrestis* and *melo* (Figure 2). A more distant accession designated Qishu Meshullash (QME), either *Cucumis trigonus* or *C. colossus*, both of which have been included in *agrestis* by Endl et al. [19], was included in the GBS analysis as an outlier. The smaller cluster I consists entirely of the subsp. *agrestis* accessions of the Chinensis, Makuwa, and Conomon Groups. Within the Conomon Group, accessions FRC and TOG are weakly separated from the accessions of the Chinensis and Makuwa Groups, which are interspersed among themselves. The Momordica and Duda’im accessions are distinct from the *agrestis* subspecies but are also separated from the rest of cluster II, which contains accessions of subsp. *melo* and consists of a number of sub-clusters (Figure 2). Ten accessions of the Inodorus Group form a sub-cluster IIa1, which is distinct from, but closely related to, sub-cluster IIa2 comprising the Kirkagac-type Inodorus accessions and the two Ananas-type Ameri Group accessions. The Flexuosus Group accessions (IIa3) also Group together and are most closely related to the Inodorus and Ameri accessions. An additional cluster (IIb) includes both the green-fleshed Ha‘Ogen-type Cantalupensis accessions (IIb1) and the closely related, yet distinct, green-fleshed Galia-type Reticulatus (IIb2). A third cluster IIc consists of two sub-clusters of orange-fleshed cultivars, one of which consists of the five orange-fleshed American accessions of the Reticulatus Group (IIc1) and, the other of four European accessions of the orange-fleshed Charentais-type Cantalupensis Group. The most unexpected placement was that of BEL, which we assumed to be a Charentais-type Cantalupensis melon. However, it was genetically distinct from the other subspecies melo types in clades IIa,b,c. 

The seven accessions, which were solely characterized metabolomically, are PI2015801, STA, PI435288, BSK, PMR, PI149169, and CRE, and these were classified based on 116 informative SNPs as shown in Figure S1 and Table S2. PI2015801 is a Kirkagac type of Inodorus and the PCR-based sequencing placed it near the Kirkagac-type PI334107. CRE is a unique orange-fleshed Inodorus accession and was genotypically closer to the two Kirkagac Inodorus accessions than to the more typical Inodorus accessions, HDG and PSR. BSK was classed together with the two other Conomon accessions, FRC and OHG. PI435288 has the appearance of a characteristic long-fruited Flexuosus type (Figure 1) and was genotypically classed as being closely related to the FAQ variety. PMR was most similar to the American netted Reticulatus accessions, both in appearance and based on the genetic polymorphisms. STA and PI149169 classed closely together, and related to PI435288 and FAQ.

### 2.2. Combined Analysis of All Metabolomic and Elemental Data

We analyzed fruit flesh and rind samples of the 51 melon accessions (Table 1). An independent duplicate of one accession, namely subsp. *melo* Duda’im (DUD), was included for control purposes, i.e., for internal validation of profile similarity.

A total of >80,000 molecular features were collected from the combination of nine analytical profiling strategies applied to the samples of melon flesh and rind, yielding more than 36,000 and 46,000 features for the flesh and rind, respectively (Table 2). The vast majority of features were collected by the two non-targeted MS-TOF techniques, UPLC profiling of semi-polar metabolites, and GC profiling of a polar fraction enriched for primary metabolites, including taste-relevant sugars and organic acids. For the metabolic classification purpose of this study, these molecular features remained non-annotated. However, for the selection of molecular features that were relevant to build a classification model of melon accessions (refer to Section 2.4.), we used a subset of annotated features [27]. In addition, three global variables of fruit quality were included, percent dry matter (%DM), total soluble solids (TSS), and pH, with mean values of 11.5 %DM, 9.7 °Brix, and pH 5.9, These showed considerable variability among the accessions as illustrated by their coefficients of variation of 10.4%, 26.2%, and 11.0%, respectively (*n* = 52, Table S1, Figure S2).

We combined the data sets of the profiled molecular features (Table 2) after 0.1–0.9 quantile range normalization of each of the sub-datasets, separately. Unique features of melon accessions were given high weight in our analyses through missing value substitution by zero. We combined all normalized sub-datasets into a single matrix of melon accessions characterized by all available molecular features of both flesh and rind samples. 

**Figure 3:**
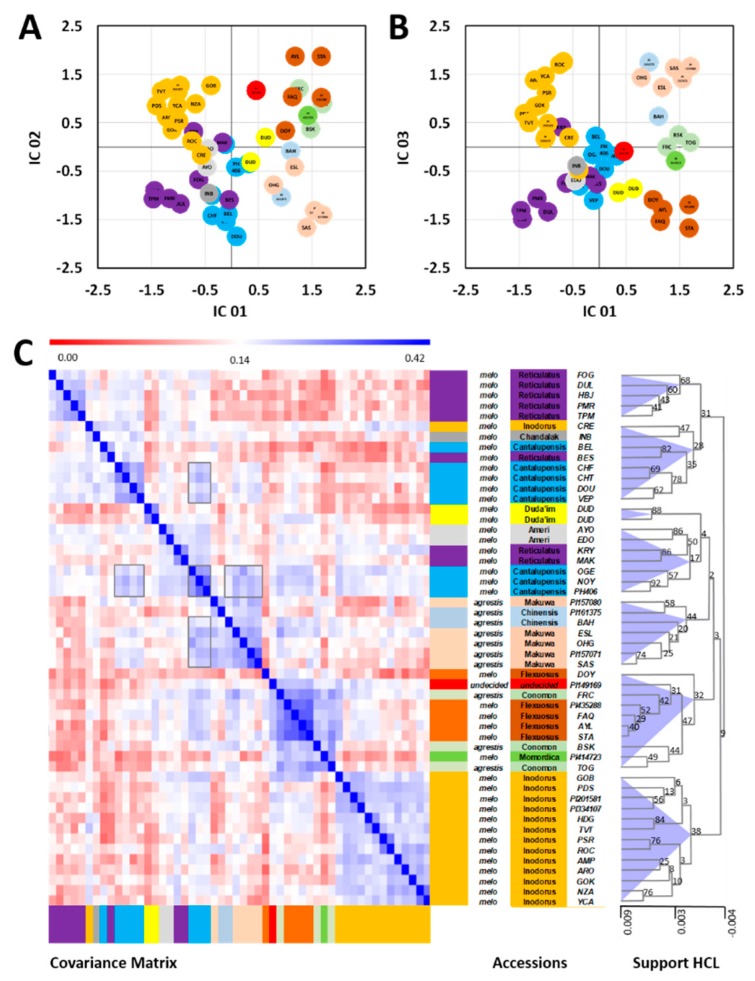
Metabolic classification of *Cucumis melo* accessions using the combined dataset of melon flesh and rind analyzed by multiplexed metabolome and elemental profiling. (**A**,**B**) ICA of the first three PCs obtained from the complete data set of >80,000 molecular features. The first three PCs comprised 23.3% of the total variance of the data set. (**C**) Covariance matrix of the complete molecular profiles of *C. melo* accessions, with HCA of the matrix using covariance distance metrics and complete linkage for clustering. HCA included support analysis by bootstrapping with 1000 iterations. Bootstrap support values of all HCA nodes and a scale of node height are included. Boxed regions of the covariance matrix indicate examples of additional alternative metabolic associations described in detail in the text. Cluster cones were arbitrarily set at the 75% tree height.

Preliminary analyses by principal components analyses (PCA) and independent components analyses (ICA) verified the complex nature of the combined dataset and revealed that only 23.3% of the total variance was represented by the first three principal components. ICA of the first three principal components (Figure 3A,B) relaxed the orthogonality criterion of PCA and instead optimized independent component kurtosis as a measure of bi- or multi-modality of the scores’ values of the resulting ICA axes [28]. The ICA scores plot array indicated separate clusters of subsp. *agrestis* and subsp. *melo*, with full or partial separation of respective accession Groups.

To transform the multi-dimensional ICA clusters of melon accessions into a mono-dimensional tree structure comparable to conventional genetic distance representations, e.g., by phylogenic or genetic distance trees, we created a covariance matrix of the complete accession profiles. For this purpose, we did not preselect features and did not apply dimension reduction methods to consider the complete molecular variance reflected by the data set. The covariance matrix was subsequently subjected to hierarchical cluster analysis (HCA) and bootstrapped to generate an HCA support tree (Figure 3C), with the node confidence validated by the bootstrap values. These values ranged from 2 to as high as 92 on a scale of 0–100. High bootstrap support was given to the terminal nodes of similar melon accessions with two of the Duda´im profiles giving an estimate of a bootstrap threshold of 82 that was indicative of the level of metabolic similarity of genetically identical melon accessions. The basal nodes of the HCA tree, however, received low bootstrap support. These results indicated that alternative basal classifications may be present in the data set. 

This interpretation was supported by alternative highly correlated groups of melon accessions that became apparent in the covariance matrix (Figure 3C). Multiple instances of alternative associations were apparent. One example was provided by the accessions of the Cantalupensis Group. This Group split between two HCA sub-branches, but alternative associations of the two Cantalupensis splits were also detected. In one HCA branch, the Cantalupensis accessions OGE, NOY, and PH406 clustered with the Reticulatus accessions KRY and MAK and the Ameri accessions AYO and EDO. In the second HCA branch, the remaining Cantalupensis accessions clustered with Inodorus accession CRE, Khandalak INB, and Reticulatus BES. 

We concluded that the reduction of all paired metabolic similarities into a tree structure perhaps only reflects in part the complex metabolic similarities that were generated by the melon breeding process. This breeding process may have included crossbreeding beyond the Group or subspecies boundaries within *Cucumis melo*. Such breeding events may cause genetic introgressions with transfers of large gene sets. In addition, breeding for metabolic quality traits may have caused partial convergence of metabolic profiles from genetically distant accessions. We found a matrix representation of accession similarity more adequate in representing a complex intra-species breeding history. Consequently, we took this approach to also compare and correlate metabolic to genetic distance matrices instead of analyzing tree similarities only (Figures S3 and S4).

The highly supported nodes of our current HCA tree (Figure 3C) were useful to evaluate the closest metabolic neighbors among the GBS-characterized and the seven PCR-characterized accessions (Table 1). The Conomon cultivar BSK (Black Skin) of subsp. *agrestis* grouped at a medium bootstrap support of 44 with TOG, a Conomon Group accession of subsp. *agrestis*. A higher-level metabolic cluster with a bootstrap support of 32 contained all five Flexuosus accessions of subsp. *melo*, including non-characterized accession STA, PI435288, and accession PI149169 of undecided Group affiliation. A well-supported cluster consistently contained 13 Inodorus accessions. This cluster included both Kirkagac-type accessions, PI201581 and PI334107, and their closest neighbor, GOB. As reported above, non-characterized CRE, provisionally classified as Inodorus, grouped to a high-level cluster of diverse subsp. *melo* accessions and was metabolically most similar to INB of the Khandalak Group. Finally, PMR membership to the Reticulatus Group was confirmed with nearest neighbor TPM (Figure 3C). PMR and TPM had high similarity to a highly supported (bootstrap value 68) cluster containing three other Reticulatus accessions.

### 2.3. Platform-Specific Metabolomic Analyses of Melon Flesh or Rind Tissue

For each analytical strategy, an HCA of accession samples was separately performed, based on the range-normalized levels of all features detected per strategy (Table 2). This HCA was performed for both the flesh (eight technological platforms, Figure S3) and rind (six technological platforms, Figure S4) of the fruits, and the similarity of the grouping of the 52 accession samples based on the observed variation in their metabolome or element composition was compared to the results based on the variation in their genome (Figure 2, Table S3). 

For flesh samples (Figure S3), the HCA showed a partial or complete clustering, depending on the analytical strategy, of accessions from the Inodorus Group. Likewise, the accessions of the Flexuosus Group were closely clustered, except for data from the NMR profiling and volatiles SPME GC-MS platforms. Grouping of Cantaloupe accessions was also clear, with the four analytical strategies detecting specialized metabolites (semi-polar extracts and volatiles) as well as for ^1^H-NMR fingerprints of polar extracts and the mineral element platform. Overall, grouping was less clear for the three platforms, detecting mainly primary metabolites (polar extracts). The grouping of accessions based on metabolites detected by SPME-GCMS and especially LC-QTOF-MS mostly showed a relatively high correlation with the genetic distances between accessions, while grouping based on primary metabolites targeted by NMR profiling data showed a relatively lower correlation. For rind samples (Figure S4), the HCA showed a partial or complete clustering of accessions from the Inodorous Group for all six platforms, except for LC-QTOF MS of semi-polar extracts for which distances within and between clusters were similar. Grouping of Cantaloupe accessions was also clear with GC-MS of polar extracts, except for one accession. 

For the 44 accessions having both GBS and metabolome or elemental data, the association between the genetic distance matrix and each metabolomic or elemental distance matrix was measured using a Mantel test, separately for flesh and rind data (Table 3). Among the 14 associations between genetic and metabolomic/elemental distances that could be tested, namely 8 for flesh, and 6 for rind (volatiles and microelements were not measured in rind), 13 including the flesh microelement-based matrix, showed a significant association with a *p*-value <0.0001. Only the metabolomic distance matrix based on LC-QTOF-MS of semi-polar extracts in rind was not related to genetic distances. For flesh, the highest two correlations between the genetic and a metabolic/elemental distance matrix were observed for specialized metabolites, i.e., for LC-QTOF-MS of semi-polar extracts (*r* = 0.56) and for SPME GC-MS of volatiles (*r* = 0.39). For rind, the highest two correlations between the genetic and metabolic/elemental distance matrix were observed for the ^1^H-NMR fingerprints of semi-polar extracts (*r* = 0.56) and for FIE-MS of semi-polar extracts (*r* = 0.47). 

### 2.4. Feature Selection by Random Forest Technology

**Figure 4:**
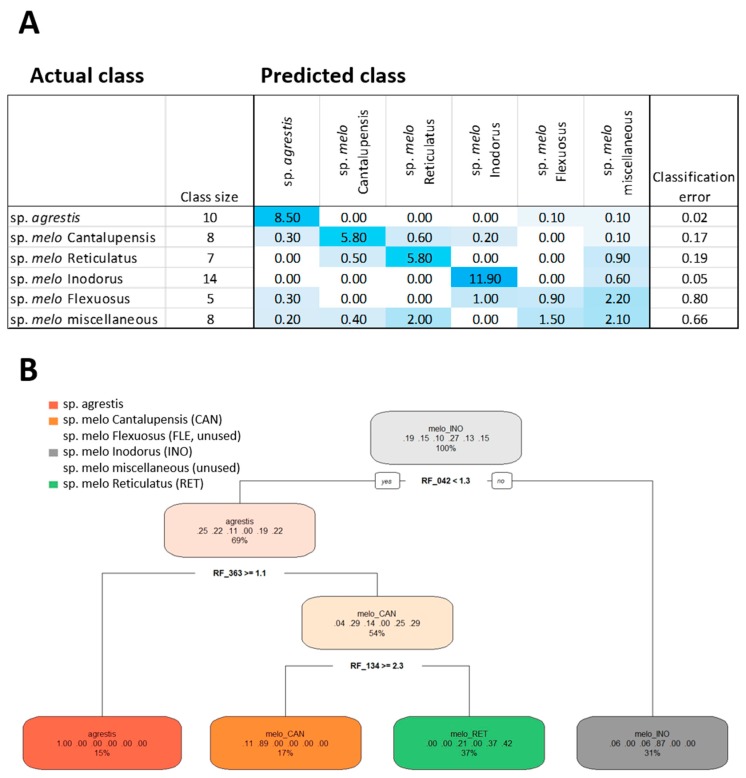
Random forest (RF) analysis (**A**) and decision tree classification (**B**) of six *C. melo* accession classes using a subset of 605 provisionally annotated molecular features. Classification of six pre-defined melon accession classes was performed. The classification table (A) lists classes, class size, the actual and predicted class membership, and the classification error (means of 10 iterations using hyperparameter-tuned RF settings). The decision tree uses the top 20 most informative molecular features ranked by the mean decrease in accuracy. The node information of the decision tree reports the used molecular feature code (Table S4) and threshold value. The branch information (colored ovals) lists the main class, the fraction of classified samples, left to right, subsp. *agrestis*, Cantalupensis, Flexuosus, Inodorus, miscellaneous, and Reticulatus accessions of subsp. *melo*. The percentage value indicates the fraction of the 52 accession samples that fall into each of the diagnostic categories.

To select molecular features that were relevant for the metabolic classification of melon accessions, we applied random forest (RF) machine learning technology. RF technology tuned towards metabolic feature selection enables the selection of small sets of molecular features that, if manually supervised, can be relevant for sample classification [29]. For this purpose, we used the set of 605 provisionally annotated molecular features from the combined >80,000 data set (Table S4). To create approximately balanced classes, we split the available melon accessions into six subsets, namely 1) all accessions of subsp. *agrestis*, 2) the Cantalupensis accessions, 3) the Flexuosus accessions, 4) the Inodorus accessions, 5) the Reticulatus accessions of subsp. *melo*, respectively, and 6) a class that contained all other melon Groups that were represented by only one or two accessions (Figure 4A). Because of the diverse nature of the sixth class, we did not expect good classification results, but we added this class to contrast with the remaining more populated classes that had 5–14 members (Figure 4).

Hyperparameter tuning of the RF procedure was applied to the six melon classes and based on the annotated data subset, provided the parameter settings: Sampled fraction of 0.899 for RF-classification, minimal node size of 2, and mtry value of 59. The estimate of the overall error rate across 10 repeated RF classifications was 23.9% ± 2.9 %, mean ± standard deviation (SD). The classification error differed, however, between tested classes. Subsp. *agrestis* classified best, with an average classification error of 0.02, followed by subsp. *melo* Inodorus with an error of 0.05. Subsp. *melo* Cantalupensis and subsp. *melo* Reticulatus classified with errors of 0.17 and 0.19, respectively. Unexpectedly, Flexuosus accessions were difficult to classify using the annotated metabolites. Molecular features relevant for the classification of the Flexuosus Group may be present among the non-annotated features (Figure 3B,C). Using annotated molecular features, the Flexuosus classification failed and had a high classification error, similar to the class of miscellaneous accessions (Figure 4A). 

The annotated mass features were ranked by RF analysis according to the variable selection parameter mean decrease in accuracy (Table S4). A decision tree based on the top 20 ranks of annotated mass features used only three features (Figure 4B). This decision tree had four classes but failed to define a diagnostic rule for the Flexuosus and miscellaneous classes. One decision rule defined a class that only contained subsp. *agrestis* accessions to 100%. A second class defined Cantalupensis accessions to 89% with a minor contribution of subsp. *agrestis* accessions (11%). A third class contained 87% Inodorus accessions with 6% each of subsp. *agrestis* and Flexuosus. The fourth class was, with 42%, enriched for Reticulatus accessions but contained, in addition, 21% Flexuosus and 37% miscellaneous accessions. The top 20 informative molecular features according to RF analysis were glucose, formic acid, glycerol, arabitol, galactinol, and raffinose in rind; xylose and 1-kestose in flesh; 10 volatile organic compounds detected in melon flesh; and Cr and As elements detected in melon flesh (Table S4). These features provide additional supporting information for melon classification.

## 3. Discussion

### 3.1. Phylogenomic and Phytochemical Relationships Partly Coincide in C. melo

Besides contributing to the genetic classification of the species, the goal of this research was to determine whether phylogenomic and phytochemical relationships in melon coincide and are mutually supportive. Studies of this nature have been carried out for over 50 years but have been limited in two significant respects. Firstly, due to the limiting nature of the targeted chemical analyses performed in earlier studies, chemosystematics was limited to interspecific classification where biochemical differences were large. Thus, interspecific chemosystematics, in conjunction with genomic classification were successfully implemented to distinguish, for example, among *Capsicum* (pepper) species [30], *Brassica* species [31], and *Citrus* species [32]. However, large-scale infraspecific classification based on chemosystematics has not been successful to date. Secondly, the comprehensiveness of metabolomic analyses, as is presented here, was made possible only recently, and earlier studies attempting chemosystematics focused on only single metabolite families, such as volatiles and carotenoids [26]. This inevitably not only limited the breadth of the characterization but also could cause strong bias in the results, especially if the metabolites most impacting the clustering have also been of selective value, either through natural selection or human selection via breeding. The variability of select metabolite families is generally controlled by few genes, which are strongly affected by selection pressure. Hence, for crop varieties, targeted analyses of metabolite variation may not reflect the genetic distance. For example, the impact of a trait, such as non-climacteric ripening, although determined by few genes, is expected to have wide-ranging pleiotropic effects on numerous secondary metabolites, including volatiles [33]. 

Our study, which is based on the combination of large-scale unbiased genetic and similarly broad characterization of metabolomic phenotypes, allowed us to determine the degree of coincidence between the phylogenetic and chemosystematic classification of the *C. melo* species. We expected to have improved representation of phylogenetic/genomic relationships in our data set by including non-targeted metabolomic technologies, as compared to a multi-targeted approach that may cover predominantly those metabolic traits that were directly subject or linked to the selective breeding process. Human breeding in melon largely aims for metabolic traits and therefore, breeding can lead to convergence of such traits in cultivars of different genotypic backgrounds and evolutionary histories. We hypothesized that non-targeted metabolites, such as those detectable by the UPLC-TOF-MS and GC-TOF-MS technologies, were presumably of less selective value during domestication and breeding than the targeted metabolites of volatiles, sugar, and organic acid levels, which together comprise the major determinants of quality and hence selection. We therefore predicted that our broad non-targeted profiling would cover the potentially convergent breeding traits, but these would be diluted by metabolic and elemental traits that were not targeted by breeding and hence hypothesized to have higher discriminatory power. Thus, chemosystematics based on non-targeted metabolites would be more likely to reflect true genetic relationships.

Systemization of melon variation goes back at least as far as the 1832 monograph of Jacquin [34]. These early classifications and nearly all subsequent ones were based primarily on fruit traits. The introduction of molecular genetic technologies has helped greatly in evaluating prospective infraspecific relationships. For example, the infraspecific classification proposed for the extremely variable *Cucurbita pepo* L. (Cucurbitaceae) that was based mainly on fruit shape [35] was supported and clarified by polymorphisms in 134 simple sequence repeat loci [36]. Rapid development of new technologies has allowed for successively and increasingly more comprehensive and precise identification and analyses of genetic variability. Most recently, high throughput sequencing and massive GBS strategies have been applied to melon variability in representative GWAS populations [8,37], yielding 17,000 and 22,000 genetic polymorphisms, respectively. Such large-scale genotyping allows for a more precise assessment of genetic relationships within species. The genotype classification of 44 of the 51 accessions used in this study was derived from the GWAS GBS previously reported by Gur et al. [37]. The remaining accessions not included in that GWAS study but screened metabolomically in this study could be reliably placed within Groups based on an additional smaller set of polymorphisms identified by direct PCR product sequencing.

Our results present a mixed picture; on the one hand the metabolomics-based infraspecific classification indeed largely reflects the phylogenetic classification. The correlation between genetic and metabolomics distance increases with the addition of metabolite signals, as expected, and the >80,000 combined metabolite signals best mirror the genetic classification (Figure 3). However, on the other hand, there are exceptions to a straightforward correlation, as will be discussed here further, and the metabolomics-based classification at times may better reflect the market-type classification rather than the genotype classification. This may indicate that selection and breeding for particular fruit characteristics may have had a disproportional impact on metabolites for which breeding did not directly select.

The present phylogenetic results substantiate the dichotomy within *Cucumis melo* to subsp. *agrestis* and subsp. *melo* (Figure 2). Furthermore, the results suggest that only the East Asian cultivar-groupGroups, Chinensis, Makuwa, and Conomon, belong to subsp. *agrestis*. The Duda’im and Momordica accessions that were included in our study are allied with subsp. *melo* but form an outlying cluster within that subspecies, as recently suggested [8,17,19]. However, with respect to the comparison of phylogenetic and the combined metabolomics-based classification (Figure 2 and Figure 3), the *agrestis* subspecies does not behave as a single Group. The two Chinensis and five Makuwa types of the *agrestis* subspecies are similar to each other based on their metabolite composition and clearly clade together. However, they are distinct metabolomically from the other *agrestis* subspecies Group Conomon. Instead, the three Conomon accessions, BSK, FRC, and TOG, clade with the Flexuosus Group, along with the single Momordica accession PI414723 and the undecided accession PI149169. Thus, the Flexuosus and Conomon accessions are metabolically more similar to each other than would be expected based on their genetic relationships. Fruits of both Groups are used primarily when young, either fresh or pickled, similar to cucumbers. The mature fruit of both Groups are characterized by an acidic pH and low sugar content, in contrast to the dessert melons with low acidity and high sugar content in the edible mature fruit [38]. In light of the fact that these two traits are governed by only a few genes [38,39,40], we expected that the large-scale metabolomics studies would overcome the bias of these few targeted metabolites and would more closely reflect the unbiased genetic relationships combining the three *agrestis* Groups. Our results with respect to *agrestis* suggest otherwise.

### 3.2. Cantalupensis and Reticulatus Accessions are Separated from Each Other Both Genotypically and Metabolomically

With regard to the Cantalupensis and Reticulatus accessions, the combined metabolomics-based classification does strongly parallel the phylogenetic classification. The separation of Reticulatus/Cantalupensis into two individual clades, IIb and IIc (Figure 2), that we observed in the genetic cladogram is similarly evident in the metabolomics classification (Figure 3). The Reticulatus Galia types (MAK, KRY) and the Cantalupensis Ha‘Ogen types (OGE, NY, PH406) have a distinct metabolite profile from the Reticulatus/Cantalupensis Groups of American cantaloupes and Charantais. Furthermore, within each of the two Reticulatus/Cantalupensis Groups, the Cantalupensis and Reticulatus accessions are separated from each other, both genotypically and metabolomically. This is evident in the separation between the Ha‘Ogen and Galia types, as well as the American cantaloupe and Charentais types from each other. The Ananas-type Ameri accessions, EDO and AYO, group with the Galia and Ha‘Ogen accessions, as they did in the phylogenetic classification. Thus, the results relating to the climacteric, netted, and semi-netted *melo* subspecies (Reticulatus/Cantalupensis/Ameri) do support the similarity between genetic and metabolomics-based classification.

The Cantalupensis and Reticulatus Groups are horticulturally distinguished largely according to the extent of rind netting or reticulation, which is actually a continuous trait rather than two distinct phenotypes of netted versus smooth. The trait of rind reticulation is presumed to be relatively simply inherited [39,41,42], but there remains the possibility that the netting trait, present in the different clades of Reticulatus/Cantalupensis, may be under separate genetic control. The most visual distinction between the different clades of Reticulatus/Cantalupensis is the flesh color. While the Charentais-type Cantalupensis are orange-fleshed, the Ha‘Ogen-type Cantalupensis are green-fleshed. Similarly, the American cantaloupe Reticulatus varieties are orange-fleshed while the Galia Reticulatus are green-fleshed. Flesh color is also determined by relatively few genes [40,43,44,45], but the associated pleiotropic metabolomic effects due to chloroplast to chromoplast transformations are expected to be large.

### 3.3. The Metabolomic-Based Classification May Indicate That Two Independent Evolutionary Events Led to Non-Climacteric Ripening

The results of the GC-MS analysis of melon volatiles (Figure S3) indeed showed that volatile metabolites alone distinguish between the non-climacteric and climacteric groups. However, our comprehensive genomic and metabolomic results also indicate that the Inodorus Group is genetically distant from the other cultivar-groups and this large genetic distance cannot be attributed only to the limited climacteric-related genetic differences but also primarily to other unrelated genetic evolution.

Concerning the Inodorus Group, most accessions are clearly sorted on the basis of their combined metabolites, similar to their phylogenetic classification. The inclusion of the Kirkagac Inodorus accessions clearly within the Inodorus metabolite group even though they were genetically distinct from it further indicates that the relationship between genetic and metabolomics-based classification is not simple, even when the latter is based on a large number of metabolic signatures as in our study. The genetic distinction between the characteristic Inodorus Group melons and non-characteristic Inodorus types referred to as European types was also noted by Leida et al. [20].

These results regarding the genetic relationships between most of the Inodorus accessions of clade IIa (Figure 2) and those of the Kirkagac accessions in clade IIb are significant as they indicate that there may have been two independent evolutionary events leading to non-climacteric ripening, one represented in the large Inodorus Group and the other more closely related to the clade IIb climacteric types. The genetic control of climacteric ripening in *C. melo* has been studied and found to be due to mutations in just a few genes [46,47]. However, these genetic studies were performed using typical Inodorus accessions as a genetic source for the non-climacteric trait and typical climacteric Cantalupensis (Charentais) accessions. The possibility remains that the genetic cause of non-climactericism in the IIa and IIb clades may not be identical and may even be due to mutations in different genes. In support of this possibility, Eduardo et al. [48] and Vegas et al. [49] reported that a supposed non-climacteric Conomon accession, crossed with the non-climacteric Inodorus PDS, yielded climacteric phenotypes, indicating independent genetic mutations. In fact, Saladie et al. [50] pointed out that classification of melon fruit ripening behavior into just two distinct types is an over-simplification, and that, in reality, there is a continuous spectrum of fruit ripening behavior. If so, further study of the relationship between the non-climacteric traits of Kirkagac and other Inodorus genotypes could broaden the genetic variability available for this important characteristic for genetically improving fruit quality by extending the harvest period as well as the shelf life of the horticultural product.

While practically all the Inodorus accessions metabolomically clade together, the CRE variety is a striking exception (Figure 3). CRE is most similar with respect to its metabolite signature to the single Khandalak accession INB, and together are most closely related to the Charentais Cantalupensis accessions. In fact, CRE is strikingly distinct phenotypically from the other Inodorus accessions, as can be seen in Figure 1. Whereas all the Inodorus lines studied are green-fleshed, as are non-climacteric Inodorus lines in general, the CRE variety is orange-fleshed and likely also climacteric. In fact, the GC-MS volatile results of flesh (Figure S4e) also distinguish between CRE and the other Inodorus lines and place CRE together with the BEL Ha‘Ogen-type Cantalupensis. The CRE accession is a Crenshaw market type, which is considered to be a hybrid derivative of the non-climacteric, green-fleshed, smooth-skinned Casaba market-type melon and the climacteric cream/orange-fleshed Persian Cantalupensis melons and thus this may explain that metabolomically, it is similar to the latter.

The different analytical strategies and over all the different types of compounds targeted (primary or specialized metabolites, mineral elements) contributed differently to the classification of melon accessions. This may be related to the fact that some metabolite classes were under breeding selection and may represent convergence of metabolic traits rather than genetic distance. The latter seems probable for major primary metabolites, measured using ^1^H-NMR or GC-MS of polar extracts in flesh and implicated in sweetness or acidity. Convergence may also be the case for different families of specialized metabolites measured using LC-MS of semi-polar extracts, such as phenolics or alkaloids with an astringent or bitter taste, or GC-MS of volatiles, such as esters and alcohols. Even mineral elements in flesh contributed to accession classification, possibly through a link with flesh acidity, for instance, for K, or metabolic links between metabolites and mineral elements as shown previously [27]. Selection may have been less stringent for rind than for flesh composition, as for five out of the six analytical strategies used for both flesh and rind analyses, the correlations between compositional distances and genetic distances were higher for rind than for flesh (Table 3).

The correlation between genetic and metabolomics distance increased with the addition of the >80,000 combined molecular features and best mirrored the genetic classification. Nevertheless, information reduction by RF analysis allowed pointing to 20 informative molecular features that differentiate several accession groups. In flesh, 10 volatiles and xylose are possibly linked with climactericity as discussed above. Of these top 10 volatiles, 6 could be reliably annotated and are well known components of melon fruit, including (Z,E)-3-hexen-ol and 1-octen-3-ol, which result from fatty acid degradation, and also two typical acetate esters (2-methyl butyl acetate and pentyl acetate) as well as alpha-ionene [27,51]. Xylose is a major non-cellulosic neutral sugar of cell walls in cucurbit fruit [52]. Fruit softening during maturation involves cell wall degradation. For cell wall-related genes, specific genes of each gene family can be categorized as totally ethylene dependent, totally ethylene independent, or partially ethylene dependent [3]. Surprisingly, two mineral elements were in the top 20 molecular features highlighted by the RF analysis. Among the six compounds highlighted in peel, glycerol may be related with the fruit surface, such as cuticular waxes [53] or suberization [54]. Galactinol and raffinose linked classification to the raffinose family and to oligosaccharides metabolism in the peel (Table S4). Galactinol and raffinose linked with the raffinose family of oligosaccharide metabolism after phloem unloading into the melon fruit may be related to fruit sweetness [55]. 

## 4. Conclusions

The main objective of this research was to determine whether classification by large-scale, non-targeted metabolomic and element profiling technologies recapitulates or extends the phylogenomic relationships within *Cucumis melo*. Our results indicate that, in general, metabolomic/elemental means of classification can indeed significantly reflect the genetic relationships. Nevertheless, there are deviations of metabolomics and elemental groupings from genetic classifications. These differences indicate that the selection for major phenotypic quality traits by the breeding process has been influential. Some of the quality traits that can be controlled by single or few enzymatic or regulatory genes, include changes of metabolite levels that define color, taste, and flavor. In addition, some traits change fruit development and thereby have pleiotropic effects on fruit metabolomics.

Large-scale information on genomic sequence and metabolomic/elemental variation of a broad range of genetic diversity can serve for dissecting the genetic basis of metabolic diversity. This process has been recently referred to as mGWAS (metabolite genome wide association study) [56]. mGWAS presents a powerful tool for attributing metabolite variation to particular genetic regions. In melon, GWAS based on GBS was even capable of mapping traits to the single candidate gene level [37] and it is expected that the comprehensive metabolomics data presented here will allow for a large-scale mapping of metabolic traits. 

Furthermore, this work sets the stage for an unlimited number of metabolic QTL (mQTL) analyses based on recombinant populations generated from selected metabolically characterized parental lines. The identification of valuable genetic and metabolic variability forms the basis for directed crop diversification and genetic improvement by breeding. The future combination of the results of this study with gene expression data of the developing melon fruit rind and flesh provides an ‘omics’ blend of genomics, metabolomics, and transcriptomics that will be especially useful for the identification of the genetic basis of metabolic diversity [44]. 

## 5. Materials and Methods 

### 5.1. Plant Material Description, Cultivation, and Sampling

An initial core collection comprised of 51 *Cucumis melo* accessions representing a broad spectrum of melon genetic variation was grown as a spring-summer crop in an open field at the Newe Ya‘ar Research Center in the Yizre‘el Valley, northern Israel. The accessions prospectively represented both subspecies and 11 of the cultivar-groups (Table 1; Figure 1; Table S1). These 51 accessions were a representative subset of a larger collection of 177 accessions that were used for detailed characterization of fruit trait variation and evaluation of the potential of genome-wide association (GWA) for trait mapping in melon [37].

Of the 51 accessions used for metabolomics analysis, 7 were not included in the GBS study but were genotypically classified in a less encompassing sequencing project based on direct sequencing of 20 PCR products of known genes. This direct sequencing comprised a total of nearly 12,000 bp and provided 116 polymorphisms for which haplotypes were produced and used for the phylogenetic analysis (Table S2). This phylogenetic analysis is not as comprehensive as the GBS but was useful in placing those accessions for which GBS data was missing.

Among these 51 accessions were included two closely related Duda’im melons (DUD2, DUD3) for a total of 52 sampled accessions. Each accession was represented in three replicated plots of five plants each. A minimum of 10 ripe fruits were harvested from each accession. Fruit were photographed prior to sampling. For sample preparation, a midsection of each fruit was excised, and its seed cavity removed. For the rind sample, the rind was removed using a vegetable peeler to a depth of approximately 2–3 mm and the rinds from all fruit of each accession were combined into a single sample. Similarly, the flesh samples of each accession were combined. Combined bulk samples were ground to a powder in liquid nitrogen. Falcon tubes were filled with approximately 50 mL of powdered sample and a total of 104 tubes (flesh and rind samples of 52 accessions) were stored at −80 °C until shipment under dry ice to each of the participating laboratories where two replicate samples were analyzed per accession/per platform.

### 5.2. Genotype and Phylogenetic Analysis

#### 5.2.1. DNA Isolation for Genotype by Sequencing (GBS)

For the GBS of the melon collection, leaf tissue was taken from 44 of the 51 accessions, listed in Table 1. Total genomic DNA isolation was performed as described by Gur et al. [37] using the GenEluteTM Plant Genomic DNA miniprep kit (Sigma, St. Louis, MO, USA). The quality of the DNA was analyzed with an ND-1000 spectrophotometer (Nanodrop Technologies, Wilmington, DE) and by electrophoresis on agarose gel. The concentration of DNA was estimated using a Qubit^®^ 2.0 Fluorometer (Life Technologies, City, Singapore) and a Qubit^®^ dsDNA BR Assay Kit (Life Technologies, Eugene, OR, USA).

#### 5.2.2. GBS Analysis

DNA was analyzed at the Institute for Genomic Diversity facility at Cornell University for GBS. GBS 96-plex libraries were prepared using the restriction enzyme ApeKI, following an established protocol [57]. Fragments were sequenced on an Illumina HiSeq 2500 as 100 bp, single-end reads and aligned to the reference genome of *C. melo* [58] available at https://melonomics.net/files/Genome/Melon_genome_v3.5.1/. TASSEL pipeline v3.0.173 was used for sequence alignment and single nucleotide polymorphism (SNP) calling [59]. Further filtration was performed using TASSEL v5.2.33 [60]; the SNP list was filtered to MAF > 0.05 and a maximum of 6% missing data per site.

#### 5.2.3. Phylogenetic Analysis

TASSEL software (v5.2.33) was used to estimate the distance matrix for all pairwise combinations. The phylogenetic tree based on the GBS results of the 44 accessions was assembled using the neighbor-joining function. In addition, we constructed a more limited phylogenetic tree based on 116 polymorphic sites derived from direct sequencing of 11,950 bp derived from 23 genomic sequences (Table S2). The sites were concatenated to create a pseudo-sequence haplotype, which was then aligned (Figure S1a). The aligned sequences were used to build a neighbor-joining tree, and a Phylip distance matrix (Figure S1b). The final alignment, tree, and distance matrix were performed using Clustal Omega version 3.0, www.ebi.ac.uk/Tools/msa/clustalo.

### 5.3. Global Measurements of Fruit Quality

Fruit total soluble solids (TSS, as degrees Brix) and pH were measured on a set of the frozen powdered samples. Portions were allowed to defrost, and juice was measured using a hand-held refractometer (Atago A-10) and a pH meter for each of the 52 accessions used for metabolomic and elemental measurements. Percent of dry matter (%DM) was measured by drying a representative sample in a 60 °C oven and weighing before and after. Means per accession were calculated.

### 5.4. Metabolomics and Elementals Analysis

#### 5.4.1. NMR-Based Metabolomic Analyses

For targeted ^1^HNMR, polar metabolites were extracted from 50 mg of lyophilized powder using a hot ethanol/water series, and then analyzed, identified, and quantified by ^1^H-NMR profiling as previously described [27,61]. For the preparation of extracts and NMR acquisition parameters, special care was taken to allow absolute quantification of individual metabolites. Quantitative ^1^H-NMR spectra were recorded at 500.162 MHz and 300 K on a Bruker Avance spectrometer (Wissembourg, France) with a 5-mm inverse probe using a 90° pulse angle and an electronic reference for quantification with calibration. Two replicates were extracted and analyzed for each accession. Unknown metabolites were named using the mid-value of the chemical shift and the multiplicity of the corresponding resonance group and quantified in arbitrary units. The same spectra issued from polar extracts were also processed as fingerprints: The spectra from δ9.40–0.40 ppm (without the residual water resonance) were binned to chemical shift regions of 0.04 ppm and data were scaled to the total signal intensity.

For untargeted NMR fingerprinting of semi-polar extracts, metabolite extraction was carried out according to the methods described by Ward et al. [62]. Briefly, triplicate aliquots (15 mg) of freeze-dried powder were extracted with an 80:20 D_2_O:CD_3_OD mixture (1 mL) containing *d4*-TSP as the internal standard (0.01% *w/v*). Samples were extracted for 10 min at 50 °C. Supernatants were transferred to a clean tube and heated to 90°C for 2 min. Samples were cooled and centrifuged. For ^1^H-NMR, 200 µL of the supernatant was evaporated to dryness and reconstituted in 650 µL of deuterated sodium phosphate buffer solution (pH 6.0, 200 mM). Samples were mixed and allowed to stand at room temperature for 1 h after which 600 µL were then transferred to a 5-mm NMR tube for ^1^H-NMR analysis. ^1^H-NMR spectra were collected at 600 MHz on a Bruker Avance spectrometer equipped with a 5-mm selective inverse probe. Parameters for data acquisition are as described in Ward et al. [62]. ^1^H-NMR data (δ9.395–0.505 ppm) were binned to chemical shift regions of 0.01 ppm and data were scaled to the total signal intensity. Regions corresponding to residual water and methanol were removed (H_2_O 4.775–4.865; MeOH 3.285–3.335 ppm set to zero).

#### 5.4.2. GC-MS-Based Metabolomic Analysis of Polar Compounds

GC-MS profiling analysis of a polar metabolite fraction enriched for primary metabolites, including sugars, amino acids, and organic acids, was performed as described earlier [63] with modifications reported by Moing and co-authors [27]. Samples were extracted, partitioned into a polar liquid phase, and dried for chemical derivatization, as described. Methoxymation was performed with 40 µL of pyridine containing 1 mg/mL methoxyaminohydrochloride and 80 µL silylation-mixture, containing 7:1 (*v/v*) N,O-bis(trimethylsily)-trifluoroacetamide (BSTFA, Macherey-Nagel, Düren, Germany) and a mixture of alkanes in pyridine. GC-MS was by splitless mode after injection of 1 µL of chemically derivatized sample. Evaluated mass features had intensities of ≥ 50 arbitrary intensity units. Peak heights of mass features defined by nominal mass to charge ratios (m⁄z) and *n*-alkane based retention indices were normalized to the sample fresh weight and the internal standard, succinic-*d*_4_ acid (Sigma-Aldrich, Deisenhofen, Germany). The internal standard was added to the extraction solution. Normalized mass spectral features were aligned, correlated across all recorded samples, and placed into clusters and time groups using TagFinder [64]. Metabolite annotations of mass spectral features were manually supervised using TagFinder visualizations for mass spectral matching [64]. Metabolite annotation required a match of at least three co-eluting and correlated mass features and a retention index deviation < 5% [65] compared to the reference data of the Golm Metabolome Database, http://gmd.mpimp-golm.mpg.de/ [66]. 

#### 5.4.3. GC-MS-Based Metabolomic Analysis of Volatile Compounds

For GC-MS of volatile organic compounds, headspace-solid phase micro extraction (SPME) with a 65-mm polydimethylsiloxane-divinylbenzene fiber (Supelco, Bellefonte, USA) was used as described previously [67]. In short, 200 mg of frozen powder was mixed with 4 mL of 4.6 M CaCl_2_ containing 5 mM EDTA in a 10-mL vial. Volatiles in the sample headspace were trapped for 20 min at 50 °C with agitation (CombiPAL autosampler, CTC Analytics, Switzerland) and thermally desorbed in the GC injection port for 1 min at 250 °C. A HP-5 column, 30 m x 0.25 mm ID, 1.05 μm – film thickness (Hewlett Packard, Palo Alto, CA, USA) was used to separate the volatile compounds, applying a temperature gradient from 44 to 250°C at a speed of 5 °C min^−1^. All masses from m/z 35 to m/z 400 resulting from 70 eV electron impact ionization were recorded (MD800 electron impact MS, Fisons Instruments, Milan, Italy). Metalign software [68] was used to extract and align all mass features in an untargeted manner and masses originating from the same molecule were then clustered to reconstruct the relative intensity and mass spectrum of each detected compound, using MSClust software [69]. Volatiles were putatively annotated by matching the reconstructed mass spectra of detected compounds to the electron impact mass spectral libraries of NIST (National Institute of Standards and Technology, Gaithersburg, MD, USA) and Wiley. Subsequently, for the random forest (RF) machine learning analysis on the putatively annotated data (Supplementary Table S4), the top 10 volatiles arising as potential markers were then further manually checked for matching of the retention index and mass spectra. Annotations with an RI >20 and/or a match factor <750 were reclassified as ‘unknowns’.

#### 5.4.4. FIE- or LC-MS-Based Metabolomics Analysis

For FIE-MS, 50 µL of the semi-polar extract supernatant prepared for NMR fingerprinting was diluted with 80:20 H_2_O:CH_3_OH (950 µL). Samples were analyzed using an Esquire 3000 spectrometer (Bruker Daltonics, Coventry, UK) in both positive and negative ionization modes as described previously [24]. Spectral data were exported as ASCII files containing mass-intensity pairs and automatically reduced using AMIX software version 3.9.11 (Bruker Biospin, Coventry, UK), to a single CSV file for each ionization mode, containing integrated regions of equal width (*m*/*z* width = 1). Individual signal intensities were scaled to the total intensity and m/z regions relating to *d4*-TSP and its isotope peaks were removed from the data prior to statistical analysis.

For LC-QTOF-MS, ground frozen rind and flesh tissue (0.5 g) were extracted in 1.5 mL of methanol, containing 0.1% formic acid. The samples were vortexed vigorously, sonicated for 20 min, and centrifuged for 15 min at 4400 *g*. For rind samples, the supernatant was filtered through a 0.22-µm PVDF filter directly to HPLC vials. Flesh samples were further concentrated as follows: 1 mL of supernatant was freeze-dried and resuspended in 150 µL of 75% aqueous methanol, containing 0.1% formic acid, sonicated for 20 min, and filtered through a 0.22-µm PVDF filter directly to the insert of the HPLC vial. Melon samples were injected into a UPLC-QTOF-MS (HDMS-Synapt, Waters, Manchester, UK) in negative ionization mode as in [70] with some modifications: Short 9.5-min gradient was used for metabolite separation. The linear gradient program was as follows: 100% to 90% phase A over 1 min, 90% to 75% phase A over 3 min, 75% to 55% phase A over 2 min, and 55% to 0% phase A over 0.5 min; held at 100% phase B for 1 min; and then returned to the initial conditions (100% phase A) in 1 min and conditioning at 100% phase A for 1.0 min. A divert valve (Rheodine) excluded the first 1.3 min and last 1.8 min of injection. A mixture of 15 standard compounds, injected after each 10 samples, was used for quality control. XCMS software [71] was used for peak picking and peak alignment. Intensity values were log^e^-transformed.

#### 5.4.5. Elemental Analysis

For microelements, frozen milled melon samples were freeze-dried and 200–250 mg dry material was wet digested (5 mL 65 % HNO_3_ and 5 mL 15 % H_2_O_2_) at 210 °C in 100-mL closed vessels using a microwave oven (Multiwave 3000 software version 1.24, Anton Paar, Graz, Austria). Before analysis, the digests were diluted to a final concentration of 3.5% HNO_3_. Multi-element analysis was performed using an ICP-MS (Agilent 7500ce, Agilent Technologies, Wokingham, UK) as described in Bernillon et al. [24]. The impact of spectral interferences was reduced using an octopole ion guide, pressurized with He or H_2_ certified reference material (NIST 1515, apple leaves, particle size <75 μm; National Institute of Standards and Technology, Gaithersburg, MD, USA) were included. Only elements deviating less than ±10 % from the certified reference values are reported.

#### 5.4.6. Data Handling and Mining

Metabolome and elemental data, i.e., the means of at least two technical replicates per accession sample and of each fruit tissue, were gathered from the consortium of laboratories. Initial response/abundance values below zero were judged to be missing data and removed. Data of each molecular feature were log_10_-transformed response ratios relative to the median across all samples. Subsets of each analytical technology, including both concatenated flesh and rind profiles, were 0.1 - 0.9 quantile range-normalized to harmonize the diverse linear response ranges of each profiling technology. Missing data were replaced by zero to give enhanced weight to subspecies- or group-specific molecular features rather than inferring values by more elaborate means, e.g., [72,73]. The resulting data set of > 80,000 molecular features was analyzed either in combined mode or divided into subsets.

Independent component analysis (ICA) of 52 samples included 51 accessions (Table 1) and a second independent sample of the Duda´im accession. ICA was applied to the first three components of a preceding principal component analysis and was performed using the MetaGeneAlyse web-application, https://metagenealyse.mpimp-golm.mpg.de/ [28]. ICA scores values were plotted. The Multi Experiment Viewer software v 4.9 [74] generated the covariance matrix of the combined data set and a support hierarchical cluster analysis (support HCA) using covariance distance, complete linkage, and bootstrapping by 1000 iterations. Bootstrap values range from 1–100 and represent the frequency of tree-node occurrence across the iterations.

In order to compare accession clustering based on genetics with the ones based on compositional distances for each tissue and each analytical strategy separately, Euclidian distances were calculated using the corresponding metabolome or ionone sub-dataset with zeros for undetected metabolomic features for the 52 accession samples with Multi Experiment viewer v 4.9. Then, accession sample clustering based on compositional data was performed under R (https://www.R-project.org/) using the Euclidian distance matrices calculated with Multi Experiment viewer and with the “complete” method for sample hierarchical clustering. In order to compare distances between accessions based on genetics with the ones based on composition, each compositional Euclidian distance matrix was restricted to the 44 accessions having both molecular and fruit metabolome or elemental data. Then, a Mantel test was done to measure the association between the molecular distance matrix and each metabolomic or elemental distance matrix by calculating the Pearson correlation between these two matrices, with its *p*-value calculated with 10000 Monte Carlo simulations using XLSTAT (v2019, Addinsoft, Paris, France). When the *p*-value was below 0.001, the two matrices were considered as significantly correlated.

To select annotated metabolic features that are relevant to classifying the *C. melo* accessions, random forest (RF) analysis was performed using the subset of annotated molecular features [27] from the combined data set (Table S4). Feature selection by RF technology was as described earlier [29] using hyperparameter optimization proposed by [75]. The R-package randomForest v 4.6–14 [76,77] was used for training classification trees. The hyperparameter tuning was performed using the tuneranger function from the tuneRanger package V 0.2 [75]. The optimized parameters were mtry, node size, and sample size. Other non-tuned RF parameter settings were ntree = 10,000, importance = TRUE, replace = TRUE. The decision tree was created by the functions rpart and rpart.plot from the rpart V 4.1–13 and rpart.plot V 2.2.0 packages, respectively, using the method “class”. We selected the top 20 molecular features according to the mean decrease of accuracy from 10 repeated RF analyses. A decision tree was created, limiting the tree to only those branches that contain all members of a class. The averaged mean decrease of accuracy and mean decrease of the Gini index were correlated by Pearson’s correlation coefficient (r²) 0.931 assuming linear association.

All data were visualized by the respective R-packages and the Multi Experiment Viewer [74] in combination with Microsoft Excel and PowerPoint.

## Figures and Tables

**Table 1:** List of melon accessions used in this study. All accessions were analyzed for metabolites. The seven accessions not included in the GBS analysis are noted as NI. Further details regarding the accessions, including classification of climacteric behavior, are presented in Table S1.

Accession Name	Subspecies	Cultivar Group	Accession Abbreviation	Included in GBS	Clade (as in Figure 2)
Batley’s Hermaphrodite	*agrestis*	Chinensis	BAH	x	I
PI161375	*agrestis*	Chinensis	PI161375	x	I
Black Skin	*agrestis*	Conomon	BSK	NI	
Freeman’s Cucumber	*agrestis*	Conomon	FRC	x	I
Tokyo Giant	*agrestis*	Conomon	TOG	x	I
Early Silver Line	*agrestis*	Makuwa	ESL	x	I
Ohgon	*agrestis*	Makuwa	OHG	x	I
PI157071	*agrestis*	Makuwa	PI157071	x	I
PI157080	*agrestis*	Makuwa	PI157080	x	I
Sakata’s Sweet	*agrestis*	Makuwa	SAS	x	I
Ananas Yoqne‘am	*melo*	Ameri	AYO	x	IIa2
Ananas ‘En Dor	*melo*	Ameri	EDO	x	IIa2
Bellegarde	*melo*	Cantalupensis	BEL	x	IIc2
Charentais Fom 2 Res.	*melo*	Cantalupensis	CHF	x	IIc2
Charentais	*melo*	Cantalupensis	CHT	x	IIc2
Doublon	*melo*	Cantalupensis	DOU	x	IIc2
Noy Yizre‘el	*melo*	Cantalupensis	NOY	x	IIb1
Ogen	*melo*	Cantalupensis	OGE	x	IIb1
PH 406	*melo*	Cantalupensis	PH406	x	IIb1
Védrantais	*melo*	Cantalupensis	VEP	x	IIc2
Indian Best	*melo*	Khandalak	INB	x	II
Duda’im	*melo*	Duda’im	DUD	x	II
PI435288	*melo*	Flexuosus	PI435288	NI	
Armenian Yard Long	*melo*	Flexuosus	AYL	x	IIa3
Doya	*melo*	Flexuosus	DOY	x	IIa3
Faqqous	*melo*	Flexuosus	FAQ	x	IIa3
Striped Armenian	*melo*	Flexuosus	STA	NI	
Amarillo Pipa Blanca	*melo*	Inodorus	AMP	x	IIa1
Amarillo Oro	*melo*	Inodorus	ARO	x	IIa1
Crenshaw	*melo*	Inodorus	CRE	NI	
Golden Beauty	*melo*	Inodorus	GOB	x	IIa2
Gold King	*melo*	Inodorus	GOK	x	IIa1
Honey Dew Green	*melo*	Inodorus	HDG	x	IIa1
Noy Zahov	*melo*	Inodorus	NZA	x	IIa1
Piel de Sapo	*melo*	Inodorus	PDS	x	IIa1
PI 334107, Kirkagac	*melo*	Inodorus	PI334107	x	IIa2
PI 201581b, Kirkagac	*melo*	Inodorus	PI201581	NI	
Piel de Sapo Redon	*melo*	Inodorus	PSR	x	IIa1
Rochet	*melo*	Inodorus	ROC	x	IIa1
Tendral Verde Tardio	*melo*	Inodorus	TVT	x	IIa1
Yellow Canary	*melo*	Inodorus	YCA	x	IIa1
PI414723	*melo*	Momordica	PI414723	x	II
Bender’s Surprise	*melo*	Reticulatus	BES	x	IIc1
Dulce	*melo*	Reticulatus	DUL	x	IIc1
Fordhook Gem	*melo*	Reticulatus	FOG	x	IIc1
Hale’s Best Jumbo	*melo*	Reticulatus	HBJ	x	IIc1
Krymka	*melo*	Reticulatus	KRY	x	IIb2
Magyar Kincs	*melo*	Reticulatus	MAK	x	IIb2
PMR45	*melo*	Reticulatus	PMR	NI	
Top Mark	*melo*	Reticulatus	TPM	x	IIc1
PI149169	undecided	undecided	PI149169	NI	
Qishu Meshullash	(outlier)	(feral)	QME	x	(outlier)

**Table 2:** Summary of the metabolome and elemental data measured using MS or NMR analytical strategies in extracts of fruit flesh or rind samples from of 52 melon accessions.

Analytical Strategies and Corresponding Examples of Covered Compounds or Compound Families	Number of Molecular Features	
	Flesh	Rind
GC-MS of polar extracts: soluble sugars, sugar-alcohols, organic acids, amino acids, polyamines	12 397	13 200
^1^H-NMR fingerprints of polar extracts: major soluble sugars, organic acids, amino acids and other amino compounds	40	28
^1^H-NMR quantitative profiles of polar extracts: major soluble sugars, organic acids, amino acids and other amino compounds	108	108
^1^H-NMR fingerprints of semi-polar extracts: major soluble sugars, organic acids, amino acids and major semi-polar specialized metabolites	839	819
DI-ESI-MS of semi-polar extracts: positive ionization mode negative ionization mode semi-polar major and specialized metabolites	931931	931931
LC-QTOF-MS of semi-polar extracts: negative ionization mode non-volatile specialized metabolites and their conjugates including the flavonoid- and hydroxycinnamate-families	20 785	30 695
SPME GC-MS of volatiles: volatile specialized metabolites including alcohols, aldehydes, terpenoids	282	-
ICP-MS of mineral elements: mineral elements including P, K, Fe, Ni, and low-abundant trace elements	20	-

**Table 3:** Link between genetic and compositional distances measured from melon fruit flesh or rind. Mantel test between the molecular distance matrix and each metabolomic or elemental distance matrix for the 44 accessions genotyped and phenotyped. ‘-‘ indicates that these analyses were not performed on the rind samples.

Analytical Strategy	GC-MS of Polar Extracts	^1^H-NMR Fingerprints of Polar Extracts (0.04 ppm VS bucketting)	^1^H-NMR Quantitative Profiles of Polar Extracts	^1^H-NMR Fingerprints of Semi-Polar Extracts (0.01 ppm bucketting)	FIE-MS of Semi-Polar Extracts	LC-QTOF-MS of Semi-Polar Extracts	SPME GC-MS of Volatiles	ICP-MS of Mineral Elements
Flesh								
Pearson correlation (*r*) between the molecular distance matrix and the metabolomic or elemental distance matrix	0.207	0.117	0.224	0.202	0.149	0.560	0.387	0.177
Correlation *p*-value ^a^	< 0.0001	< 0.0001	< 0.0001	< 0.0001	< 0.0001	< 0.0001	< 0.0001	< 0.0001
Rind								
Pearson correlation (*r*) between the molecular distance matrix and the metabolomic or elemental distance matrix ^b^	0.315	0.273	0.267	0.561	0.468	−0.041	-	-
Correlation *p*-value	< 0.0001	< 0.0001	< 0.0001	< 0.0001	< 0.0001	0.189	-	-

*^a^ p*-value calculated with 10000 Monte Carlo simulations. ^b^ Euclidian distance on range-normalized data for metabolomic or elemental distance matrices between accessions.

## Supplementary Materials

The following are available online at https://www.mdpi.com/2218-1989/10/3/121/s1, Figure S1: Phylogenetic tree and alignments based on direct PCR sequencing of melon accessions not included in GBS, Figure S2: Fruit quality global measurements of 52 accession samples, Figure S3: Hierarchical clustering analysis of 52 accession samples performed per analytical platform for fruit flesh, Figure S4: Hierarchical clustering analysis of 52 accession samples performed per analytical platform for fruit rind, Table S1: Description of the melon accessions used in this study, Table S2: Genes and primers used for PCR-based genome sequencing and polymorphism identification, Table S3: Genetic distance matrix of the 44 GBS-sequenced accessions, Table S4: List of 605 provisionally-annotated molecular features from the combined data set with assignment of variable-importance obtained by RF technology.
