# Supplementary material for: Comparative Metabolomics and Molecular Phylogenetics of Melon (Cucumis melo, Cucurbitaceae) Biodiversity
Source: Metabolites. 2020 Mar 24;10(3):121. doi: 10.3390/metabo10030121 (PMC7143154; doi:10.3390/metabo10030121)
Supplement: Supplementary file 1 [file metabolites-10-00121-s001.zip › Supplementary Figures S1 S2 S3 S4.pdf]

Title: Comparative metabolomics and molecular phylo-genetics of melon (*Cucumis melo*, Cucurbitaceae) biodiversity

Authors: Moing et al.

Journal: Metabolites

Supplementary figures, S1-S4

A)

```
CLUSTAL O(1.2.4) multiple sequence alignment


PI435288      AGCAAGCGTAAAATTTTCCAGGTAAAAAGCTAGGATCCRGTATAAACCGATATAAACGAA      60
FAQ           AGCAAGCGTTCAATTTTCCAGCTAAAGGGGTAGGATCCGRTATAGGCCGATACAAACGTG      60
PMR45         AGCAAGCGCAAAATTTTCCACCTCGTGGGCTAGGATTTAGTAAAAACCGACACAAAGGTA      60
HBJ           AAATGGCGCAAAATTTTCCACCTCGTGGGCTAGGATTTAGTAAAAACCGATATAAACGAA      60
BES           AAATGGCGCAAAATTTTCCAGCTAAAGGGCTAGGATTTAGTAAAAACCGATATAAACGAA      60
OHG           AAATGGCGCTCTATTCCTTAGCTAAAAAGCTCGGACCCGRCAAAAAYSRRTRTMWRCGAA      60
FRC           AAATGGCACTCTATTCCTTAGCTAAAAAGCTCGGACCCGRCAAAAACCGATATAAACGAA      60
BSK           AAATGGCGCTCTATTCCTTAGCTAAAAATCTCGGACCCGGCAAAAACCGATATAAACGAA      60
PI149169      TATAARYGCAMAATTTTCCACCTCGTAATCTCAAGTCYGGTGCAGACCGATACAAACGAA      60
STA           AGCAAGCGCACTATTCCTTACCTCGTGGGCTAGGATCCGGTATAGGCCGATATAAACGAA      60
CRE           AGCAAGCGCAAAATTTTCCACCTCGTGAGCTAGGATCCGGTAAAAACCGATATAAACGAA      60
PI334107      TATAAATGCAAAATTTTCCACCTCGTGAGCTAGAATCCGGTAAAAACCGATATAAACGAA      60
PI201581      TATAAATGCAAAATTTTCCACCTCGTGAGCTAGGATCCGGTAAAAACCGATATAAACGAA      60
PSR           AAATGGCGCAAAATTTTCCACCTCGTGAGCTAGGATCCGGTAAAAACCGATATAAACGAA      60
HDG           AAATGGCGCAAAATTTTCCACCTCGTGAGCTAGGATCCGGTAAAAACCGATATAAACGAA      60
                          ***    *  *       *            *             *

PI435288      ACTAGCTCAAGCGSYGWYRGTTAATAGGCCCGCCATTGACTATGWMCWWGGSTGRY  116
FAQ           ACTATTCCAAGCAGCGTCGGGTAATAGGCCCGCCATTGGTCACATCCATGGCTGAT  116
PMR45         ACTATTCCAAGCRCCGTCGGGTGATAAGCCCGCCATTGACTATGATCATCGGTGGC  116
HBJ           ACTATTCCAAGCGCCGTCGGGTRATARGCCCGCCATTGACTATGWMCWWCGGTGGC  116
BES           TCCATTCCAAGCAGCGTCGRGTRATARGCCCGCCATTGACTATGTACTTCGGTGGC  116
OHG           ACTAGCTCAATTGSCCTCAGTGAGAGGATATAATGGCAACTATGTGTAACGGTGGC  116
FRC           ACTAGCTCAATCGGCGTCAGTGAGAGGATCYGCYRKYRACTACGTGTAACGGTGGC  116
BSK           ACTAGCTCAATCGGCGTCAGTGAGAGGATCCGCCATTGACTACGTGTAACGGTGGC  116
PI149169      WCYATTCCGATCGGCGTCAGTTAATAGGCATAATGGCAACTATGTACTTCAGTGGC  116
STA           ACTAGCTCAAGCGGYGTYAGTTAATAGGCATAATGGCAACTATGTACTTCGGTGGC  116
CRE           ACTATTCCAAGCGGCGTCAGTGAGAGGATATAATGGCAACTATGATCATCGGTGGC  116
PI334107      ACTAGCTCAAGCGGCGTCAGTGAGAGGATATAATGGCAACTATGATCATCGGTGGC  116
PI201581      ACTAGCTCAAGCGGCGTCAGTGAGAGGATATAATGGCAACTATGATCATCGGTGGC  116
PSR           ACTAGCTCAAGCGGCGTCAGTGAGAGGATATAATGGCAACTATGATCATCGGTGGC  116
HDG           ACTAGCTCAAGCACCGTCGGTGAGAGGATATAATGGCAACTATGATCATCGGTGGC  116
               * *   * *                               *          **
```

B)

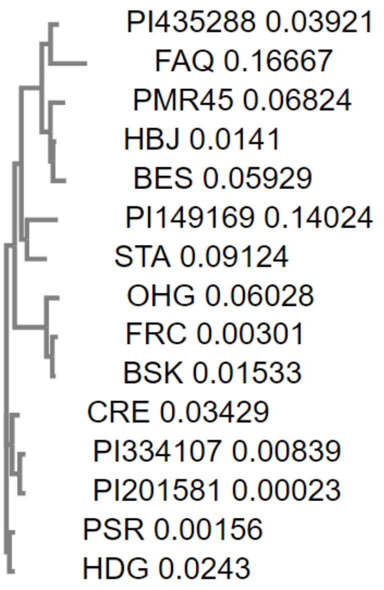

Figure S2. Classification of the seven accessions not included in the GBS analysis. A) Clustal alignment of the 116bp haplotype of the seven accessions together with GBS-analyzed closely related accessions. B) Phylogenetic tree developed by Neighbor-Joining method. Clustal Omega: https://www.ebi.ac.uk/Tools/msa/clustalo/

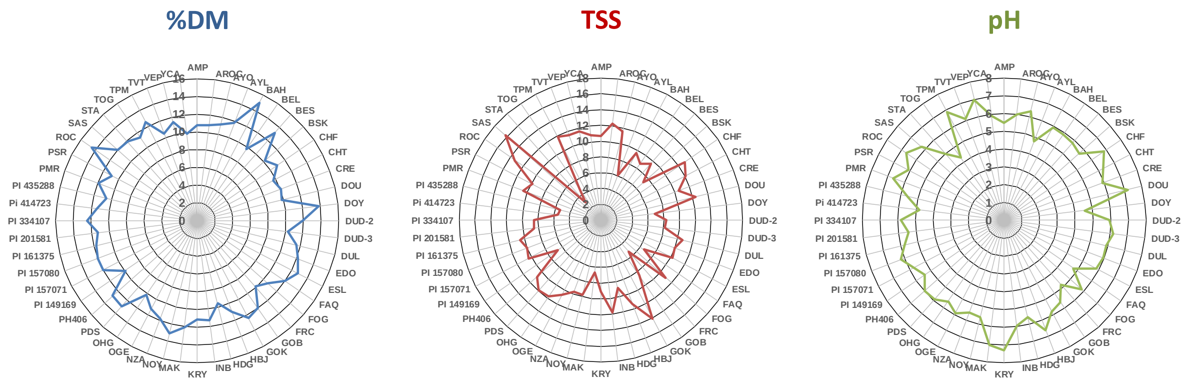

a)

Overview (n=52)	DM content (%)	TSS (°Brix)	pH
Mean	11.50	9.72	5.89
Min	9.60	3.00	4.27
Max	15.00	16.20	7.31
CV (%)	10.42	26.16	11.02

b)

Figure S2. Variability of the 52 melon accessions for fruit quality global measurements: fruit flesh percent dry matter (%DM), and fruit juice total soluble solids (TSS) and pH. CV, coefficient of variation. a) radar plots for the 52 accession samples. b) overview.

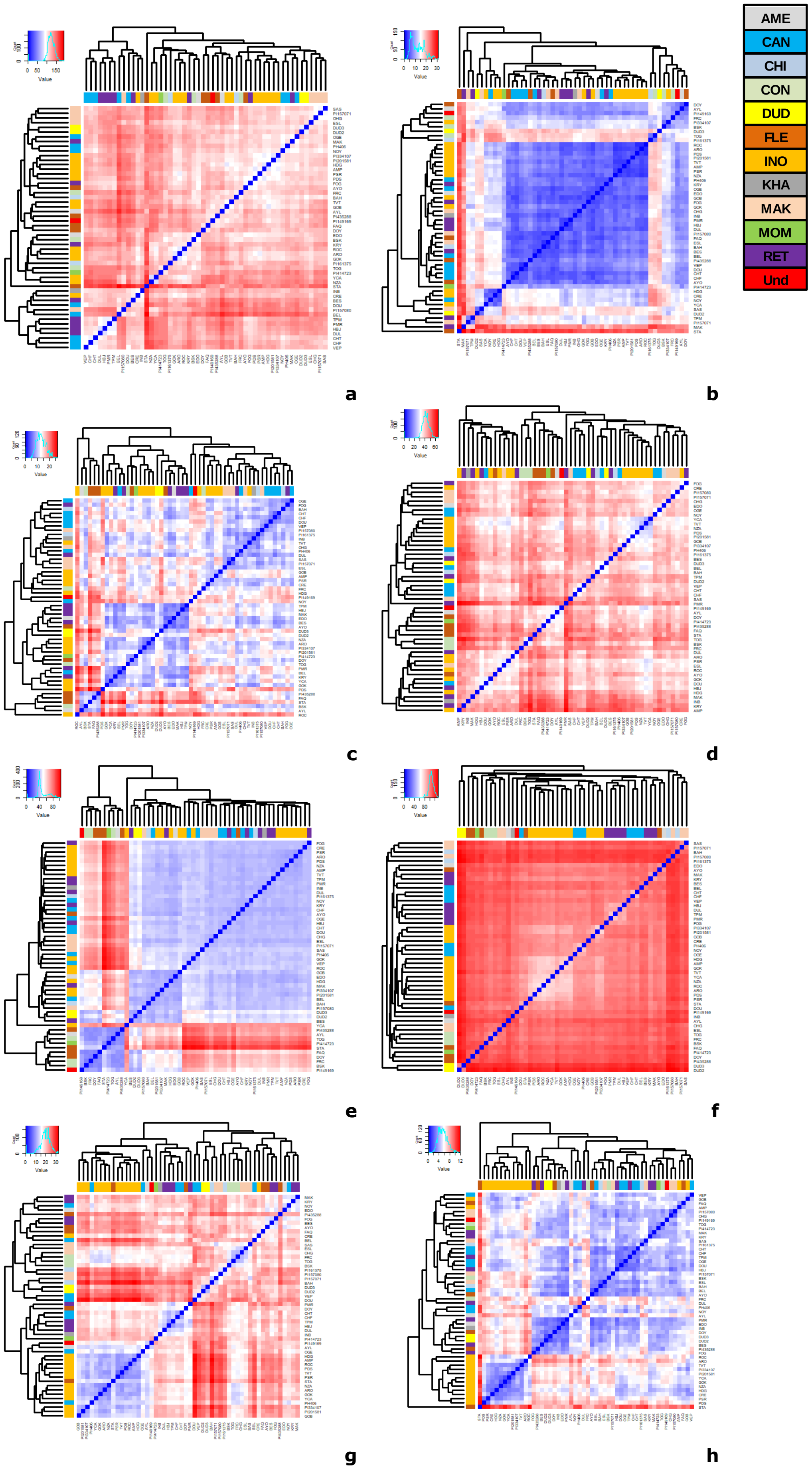
AME
CAN
CHI
CON
DUD
FLE
INO
KHA
MAK
MOM
RET
Und
a
b
c
d
e
f
g
h

Figure S3. Hierarchical clustering analysis of the 52 melon accession samples based on each analytical platform for fruit flesh with Euclidian distance and complete linkage. Each subfigure represent an accession dendrogram with heatmaps for accession x accession distances. The colored bar close to the dendrogram indicates accession groups. The distribution of distances is presented on the upper left side of each subfigure. a) GC-MS of polar extracts; b) ^{1}H-NMR profiles of polar extracts; c) ^{1}H-NMR fingerprints of polar extracts; d) ^{1}H-NMR fingerprints of semi-polar extracts; e) DI-MS of semi-polar extracts; f) LC-QTOF-MS of semi-polar extracts; g) SPME-GCMS of volatile compounds; h) ICP-MS of micronutrients.

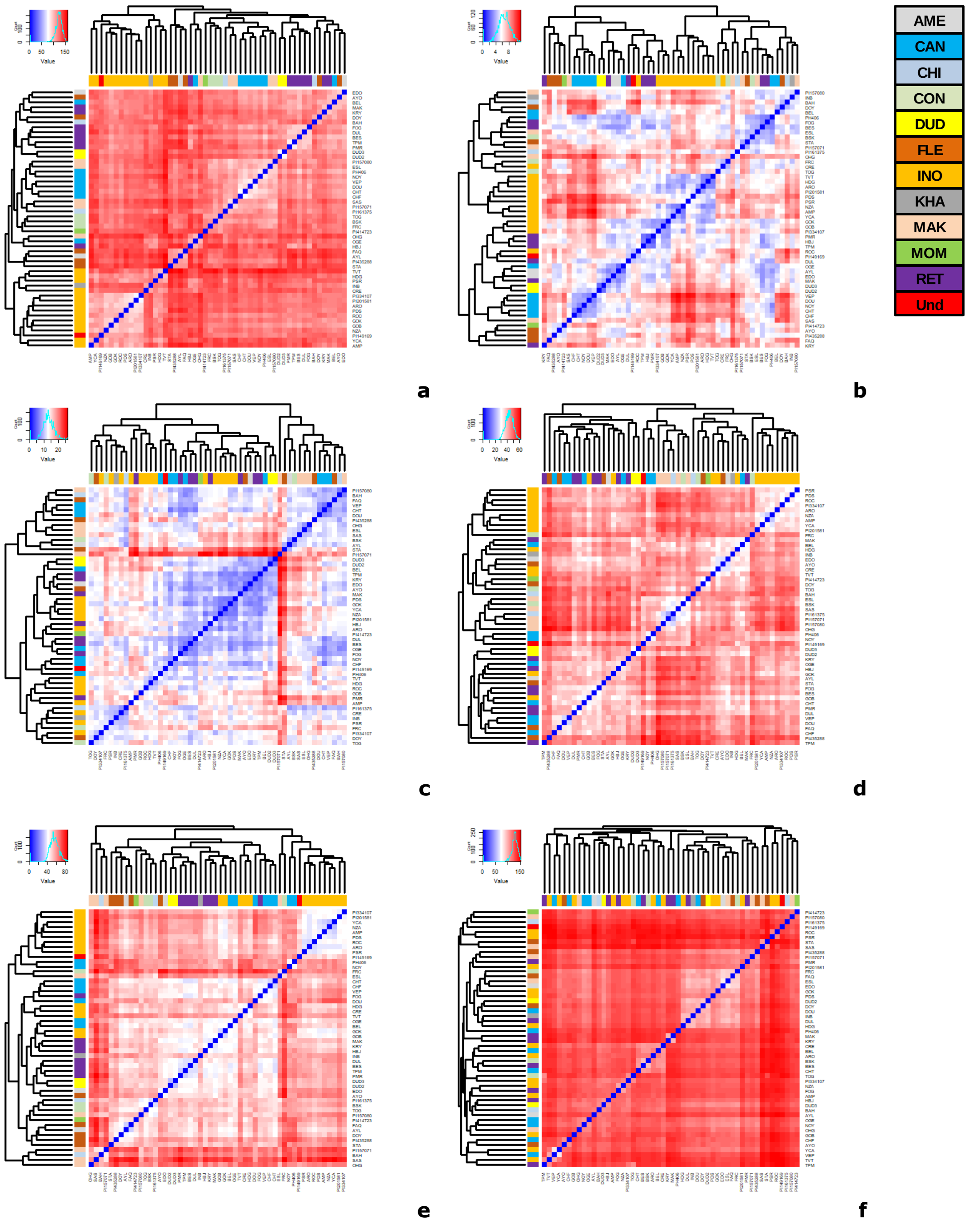

Figure S4. Hierarchical clustering analysis of the 52 melon accession samples based on each analytical platform for fruit peel with Euclidian distance and complete linkage. Each subfigure represent an accession dendrogram with heatmaps for accession x accession distances. The colored bar close to the dendrogram indicates accession groups. The distribution of distances is presented on the upper left side of each subfigure. a) GC-MS of polar extracts; b) ^{1}H-NMR profiles of polar extracts; c) ^{1}H-NMR fingerprints of polar extracts; d) ^{1}H-NMR fingerprints of semi-polar extracts; e) DI-MS of semi-polar extracts; f) LC-QTOF-MS of semi-polar extracts
